# The assessment of cortical hemodynamic responses induced by tubuloglomerular feedback using in vivo imaging

**DOI:** 10.14814/phy2.15648

**Published:** 2023-03-22

**Authors:** Blaire Lee, Dmitry D. Postnov, Charlotte M. Sørensen, Olga Sosnovtseva

**Affiliations:** ^1^ Department of Biomedical Sciences Copenhagen University Copenhagen N Denmark; ^2^ Department of Clinical Medicine Aarhus University Aarhus Denmark

**Keywords:** blood flow imaging, kidney, microcirculation, TGF

## Abstract

The tubuloglomerular feedback (TGF) mechanism modulates renal hemodynamics and glomerular filtration rate in individual nephrons. Our study aimed to evaluate the TGF‐induced vascular responses by inhibiting Na‐K‐2Cl co‐transporters and sodium‐glucose co‐transporters in rats. We assessed cortical hemodynamics with high‐resolution laser speckle contrast imaging, which enabled the evaluation of blood flow in individual microvessels and analysis of their dynamical patterns in the time‐frequency domain. We demonstrated that a systemic administration of furosemide abolishes TGF‐mediated hemodynamic responses. Furthermore, we showed that the local microcirculatory blood flow decreased, and the TGF‐induced hemodynamic oscillations were sustained but weakened after inhibiting sodium‐glucose co‐transporters in Sprague–Dawley rats.

## Graphical abstract

High‐resolution in vivo blood flow imaging was employed to unveil the TGF‐induced hemodynamics in response to systemic administration of furosemide and phlorizin in Sprague–Dawley rats. Furosemide reduced the blood flow and abolished TGF oscillations in observed microvessels as expected. Phlorizin decreased the microcirculatory blood flow and the magnitude of TGF oscillations.

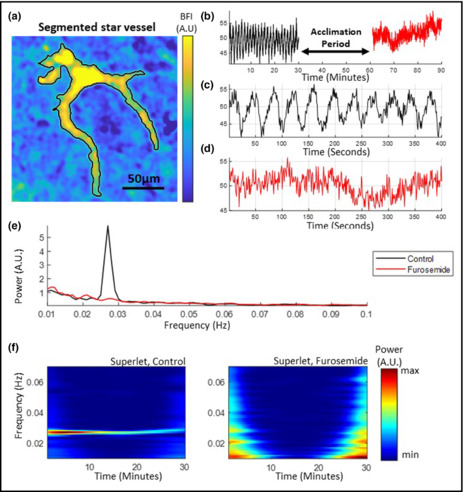

## 1 INTRODUCTION

Despite the constant fluctuation in systemic blood pressure, the kidney maintains a stable filtration rate due to renal autoregulation (Loutzenhiser et al., 2006), which takes place in individual nephrons, or structurally discernible filtration units of the kidney. The two prominent components of renal autoregulation are the myogenic response (MR) and the tubuloglomerular feedback (TGF) mechanism. While both components target the resistance of afferent arterioles, they occur in response to distinct triggers. MR is the constriction of a vessel due to increased transmural pressure. The TGF correlates to the amount of chloride passing into the distal tubule. When the macula densa detects an increase in the tubular fluid Cl^−^ concentration through the Na‐K‐2Cl co‐transporters, it signals the afferent arteriole to constrict (Briggs & Schnermann, 1987; Burke et al., 2014; Just, 2007; Schnermann, 1998). The effect is reduced blood flow and hydrostatic pressure into the glomerulus. Typically, the TGF mechanism modulates the afferent arteriolar resistance at around 0.033 Hz resulting in oscillatory blood flow. Managing glomerular capillary pressure is pertinent for the kidney to sustain function and prevent deterioration.

Since the concentration of tubular sodium chloride can modulate the TGF, pharmacologically altering the reabsorption of the ions by inhibiting various Na^+^ co‐transporters and its implications in renal autoregulation is of great interest. Furosemide is a loop diuretic for treating patients with heart failure and hypertension. Furosemide inhibits the Na‐K‐2Cl co‐transporters (NKCC2) expressed in the thick ascending limb of the loop of Henle and the macula densa, effectively blocking the reabsorption and the sensing of sodium chloride (Castrop & Schnermann, 2008; Ponto & Schoenwald, 1990). Although it is well‐known that furosemide abolishes the TGF in a single nephron (Just, 2007), the hemodynamics due to TGF inhibition has not been observed in a population of nephrons in vivo due to the lack of appropriate instruments.

The kidney plays an integral role in glucose homeostasis partly through glucose reabsorption. Sodium‐glucose co‐transporter 2 (SGLT2) expressed in the luminal membrane of the proximal convoluted tubules is responsible for 97% of the glucose reabsorption through a sodium‐dependent active transport (Vallon & Thomson, 2017). Phlorizin, an SGLT 1 and 2 inhibitor, lowers the blood glucose level, induces glucosuria, and promotes weight loss in diabetic patients by blocking the sodium‐glucose reabsorption (Vallon, 2015; White Jr, 2010). Recent findings suggest that inhibiting SLGT2 can reduce glomerular hyperfiltration and restore the TGF in a compromised kidney (Sen & Heerspink, 2021). Still, the in vivo effect of SGLT2 inhibition on nephron blood flow and the TGF remains poorly understood.

High‐resolution laser speckle contrast imaging (LSCI) can measure microvascular hemodynamics in a population of nephrons in real‐time (Lee, 2022; Postnov et al., 2022). With LSCI, one can record the microvascular blood flow at the renal surface. These microvessels, or “star vessels”, stem from the efferent arterioles and branch in a stellate fashion underneath the kidney capsule (Beeuwkes III & Bonventre, 1975; Nordsletten, 2006; Yoldas and Orhun Dayan, 2014). The LSCI can robustly detect these microvessels' TGF oscillations and nephron hemodynamics because they are anatomically downstream from the afferent arterioles, where the TGF signals originate. Yet, the approach and analysis pipeline for employing high‐resolution LSCI in acute intervention studies have not been fully established.

We acutely administered systemic infusions of furosemide (NKCC2 inhibitor) or phlorizin in anesthetized Sprague–Dawley rats. We assessed the TGF‐induced hemodynamic changes in a population of nephrons in vivo with a high‐resolution LSCI. We also applied the superlet analysis‐ an improved method for analyzing biosignals‐ to renal hemodynamics for the first time (Moca et al., 2021). We also extracted new analytical metrics to quantify the characteristics of TGF oscillations. Finally, we evaluated the effects of furosemide and phlorizin on cortical hemodynamic responses mediated by the TGF.

## 2 MATERIALS AND METHODS

### 2.1 Surgical preparation and experimental protocol

Danish National Animal Experiment Inspectorate approved all rodent experiments. Male normotensive Sprague Dawleys (*N* = 12, RjHan:SD, Janvier) weighing 330 ± 23 g were used. The animals were fed ad libitum and housed in a 12‐h light/dark cycle. Detailed animal preparation can be found in Postnov et al. (2022) and Holstein‐Rathlou et al. (2011). Here we provide an abridged version. All experiments were performed under sevoflurane anesthesia (Sevorane, Abbvie), 8% at induction, and maintained at around 1.5%. The body temperature was maintained at 37C on a servo‐controlled heating table. The animal was laid ventral side up, and a mask delivering anesthesia covered the nose and the mouth during the surgery. A small horizontal incision was made between the cervical and pectoral region to expose and isolate the trachea, the left jugular, and the right common carotid. Two polyethylene tubes (PP10, Smith Medicals) were inserted into the left jugular vein for infusions of saline (0.5% NaCl) and a muscle relaxant (0.5 mg/mL) (Nimbex, Apsen) ‐to prevent secondary breathing motion‐ at 20 μL/min via syringe pumps (AL‐1000, World Precision Instruments). A slightly larger polyethylene tube (PP50, Smith Medicals) was placed into the right carotid for continuous blood pressure monitoring. The animal was ventilated through tracheotomy with a volume‐controlled respirator (7025, Ugo Basile) at 7 mL per stroke (60 strokes/min). A laparotomy was performed to expose and stabilize the kidney with a custom 3D‐printed well. 1.5% (w/v) agarose solution was poured over the kidney, and a glass coverslip was gently placed on top of the kidney to prevent a parched surface. A myograph wire (40 μm in diameter) bent at an acute angle was placed on top of the coverslip to track visceral and breathing motion for image registration. The left ureter was cannulated with a polyethylene tube (PP10 connected to PP50, Smith Medicals) for free urine flow and sample collection. A renal flow probe (1PRB, connected to T420 Transonic) was fastened around the renal artery for an animal to measure the standard renal blood flow. Each experiment was successful for animals with a stable blood pressure of 100–130 mm Hg.

**FIGURE 1:**
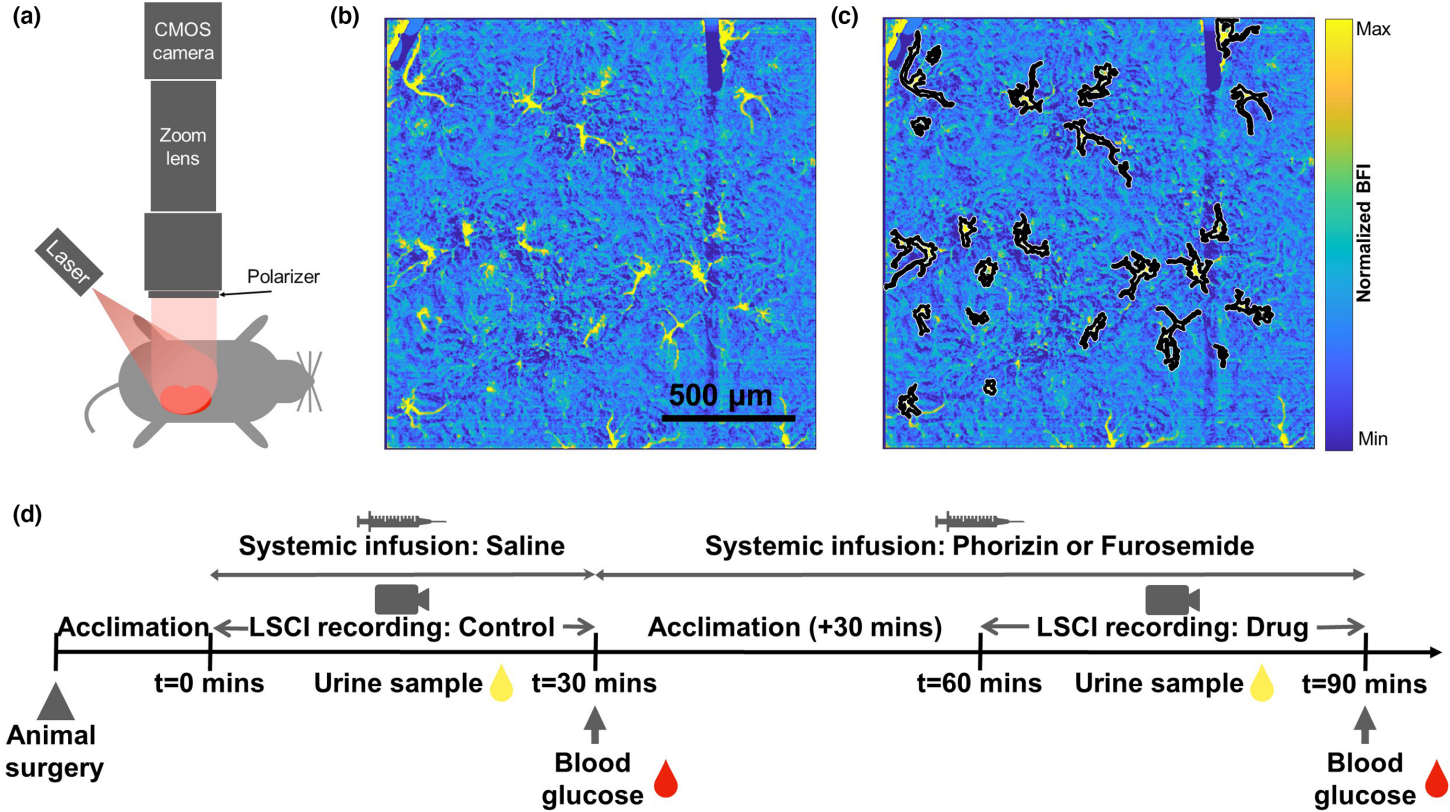
Experimental overview assessing the influence of loop diuretic (Furosemide) and Phlorizin on renal hemodynamics. (a) Imaging setup for laser speckle contrast imaging for real‐time high‐resolution tissue blood flow measurements. (b) Time‐averaged blood flow map of the renal surface acquired with the LSCI. The yellow star‐like shaped regions are microvasculatures with relatively high blood flow compared to the surrounding renal tissue. (c) The microvasculatures are segmented (demonstrated by black borders) for blood flow time series extraction. (d) The timeline for the whole experiment. The recording takes place during the drug infusion.

The animals were separated into two groups: Animals receiving furosemide (*n* = 5) and animals receiving phlorizin (*n* = 7). The experiments were carried out in an alternating pattern between the animal groups between 9 am and 5 pm. First, baseline saline infusion and image recording took place for 30 min. Then, the saline syringe connecting to the left jugular was replaced with either Phlorizin (10 mg/mL, 274,313, Sigma‐Aldrich) or furosemide (2.5 mg/mL) solution and infused at 20 μg/mL to induce glucosuria or diuresis, respectively (Malatiali et al., 2008). A minimum of 30 min of acclimation period allowed the administered substance to reach its peak systemic concentration. Finally, 30 min of postacclimation recording took place while continuing the infusion to sustain bioavailability and saturate the TGF response. The animals were sacrificed at the end of each experiment. See the experimental overview in Figure 1.

### 2.2 Imaging setup

We used a high‐resolution LSCI (Lee, 2022) to record the changes in renal blood flow with the infusion of Phlorizin or Furosemide. Here we briefly describe the imaging setup. A CMOS sensor camera (Basler acA2048‐90umNIR, 5.5 μm pixel size, 8bit mode) was vertically attached to a video zoom lens with 4.5× magnification (VZM 450, Edmund Optics). A volume holographic grating stabilized diode (LP7850‐SAV50, 785 nm Thorlabs) was used to illuminate the tissue surface and passed through a linear polarizing filter before reaching the photo‐sensors to reduce recorded specular reflections and first‐order scattering events. Finally, the apparatus was mounted to a height‐adjustable z‐stage plate to enable vertical translation. The original modality is a multiscale system, and the low zoom arm (larger field‐of‐view) can also be activated to record the whole kidney blood flow. Thirty minutes of raw speckle images were recorded at 50 Hz with 5 ms exposure time for the control and the intervention period. We eliminated all ambient light and kept all settings and system configurations consistent across every experiment.

### 2.3 Data analysis

Postacquisition data analysis approaches are described in Lee (2022) and Postnov et al. (2022). Here we provide a summary of each step.

#### 2.3.1 Image registration and speckle contrast analysis

We aligned the images to the first frame to eliminate the motion artifact from breathing in the *x–y* plane. A thin myograph wire was placed on the glass cover over the kidney as a reference mark during the experiment. The raw frames were converted to binary images, with the myograph wire and the surrounding tissue as the foreground and the background. We then calculated the geometrical transformation for each frame compared to the first frame and aligned the images based on transformation metrics.

We implemented temporal contrast analysis to preserve high spatial resolution to improve image quality and segmentation performance. First, temporal laser speckle contrast was calculated from raw data sets by $K=\frac{\sigma }{\mu }$. *σ* is the standard deviation, and *μ* the mean of the selected temporal kernel (25 frames). Following temporal contrast analysis, the data was sub‐sampled from 50 to 1 Hz and converted from the contrast images to blood flow index images by $\mathrm{BFI}=\frac{1}{K^{2}}$.

A mean spatial blood flow map was created by averaging the frames recorded over the time frame of interest: control and drug. Semiautomatic segmentation was applied to high‐zoom data to segment individual vessels from the surrounding renal tissue according to the approach described in Lee (2022) and Postnov et al. (2022).

#### 2.3.2 Time‐frequency analysis

TGF is an oscillatory signal that operates within a range of frequencies (0.018 and 0.033 Hz), and it can vary over time like many other biological oscillations (Zou et al., 2002). Therefore, time‐frequency super‐resolution (superlet) analysis was implemented to the extracted flow time series to reveal TGF oscillations (Moca et al., 2021). While there are many methods to analyze frequency components of biosignals, superlet was chosen for its exceptional performance in resolving the frequency of a signal that changes over time, a primary objective in detecting the changes in TGF frequencies over an observation period (Moca et al., 2021).

We extracted three metrics from the blood flow time series and the power spectrum. These metrics were compared between the control and the drug period.

- Metric 1 (BFI): Mean blood flow index was calculated by averaging the blood flow index time series over the control and the drug period.
- Metric 2 (Sigma). The standard deviation of TGF oscillations. Blood flow index time series were passed through a bandpass to filter of frequency between 0.015 and 0.04 Hz so that signals only contained the frequency range of interest. Then we calculated the standard deviation of filtered signals. We use this metric to measure the amplitude of TGF oscillations.
- Metric 3 (AUC). The area under the curve of the power spectrum within the TGF frequency. First, we performed trapezoidal numerical integration between 0.015 and 0.04 Hz of the individual power spectrum to find the area under the curve within the TGF frequency band. Then we drew a median line between 0.04 and 0.05 Hz for each power spectrum and calculated the area below the line within the TGF frequency band. Then the area below the median line was subtracted from the area under the curve. We use this metric to measure the significance of TGF band frequencies.

#### 2.3.3 Statistical analysis

Paired sample *t*‐test was used to compare the urine samples collected before and after administering either furosemide or phlorizin. In addition, the linear mixed‐effects model was used to compare the TGF metrics between the control and their respective furosemide or phlorizin data, adjusting for between‐subject variability. Results with *p* < 0.001 were declared significant. The central mark of box and whisker plots indicate the median, and the top and the box edges indicate the 75th and the 25th percentiles of the data.

## 3 RESULTS

### 3.1 Characterizing the TGF‐induced hemodynamics

**FIGURE 2:**
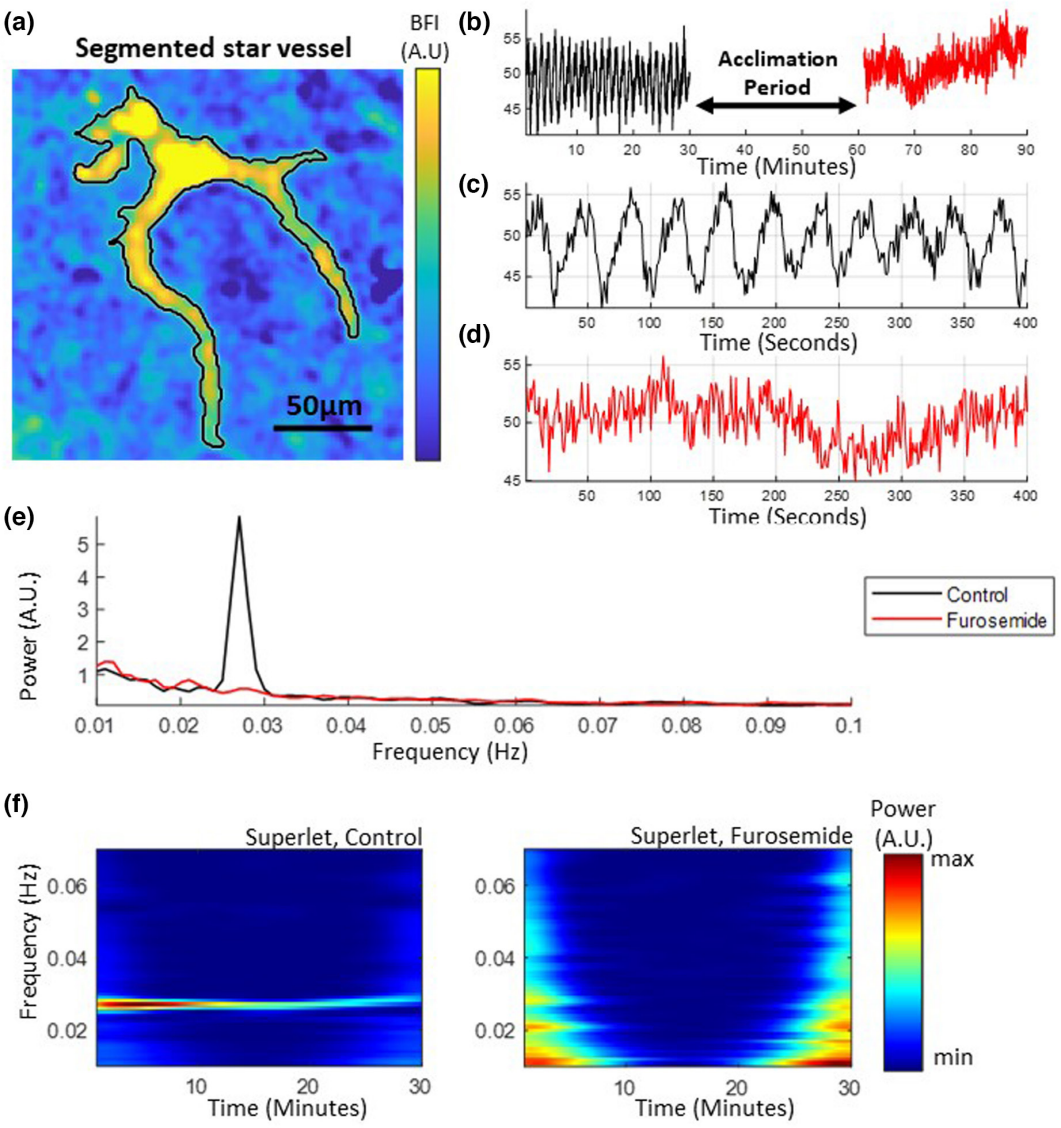
Illustration of the analysis from segmentation to a time‐frequency spectrogram. (a) A close‐up image of a star vessel that can be imaged at the renal surface. The images of vessels undergo a segmentation process, from which (b) blood flow index time series can be extracted. (c, d) A closer look at the B reveals that the TGF oscillations during the control period (black) disappear with the furosemide infusion (red). (e) Power spectrum shows a prominent peak at around 0.03 Hz associated with TGF during the control period. (f) The spectrogram of superlet analysis for the control period (left) shows a sharp localization of the TGF frequency around 0.03 Hz sustained for 30 min that disappears completely with the furosemide infusion (right).

To accurately capture the transient dynamics of TGF, we took an exemplary microvessel blood flow time series obtained from a random experiment. We decomposed the signal from the time domain to frequency‐power components using time‐frequency superlet analysis shown in Figure 2. It has been demonstrated that the superlet provides a superior time‐frequency resolution compared to the Fourier and the Wavelet transform. The time‐averaged power spectrum shows a sharp and narrow peak near 0.03 Hz, demonstrating that the superlet analysis can sharply localize the TGF frequency as shown in Figure 2e. Figure 2f shows the TGF signal over time for the control period, corresponding to the oscillation shown in Figure 2c. The line at 0.03 Hz disappears on the right panel of Figure 2f because the TGF oscillation no longer exists, demonstrated by the noisy signal in Figure 2d.

**FIGURE 3:**
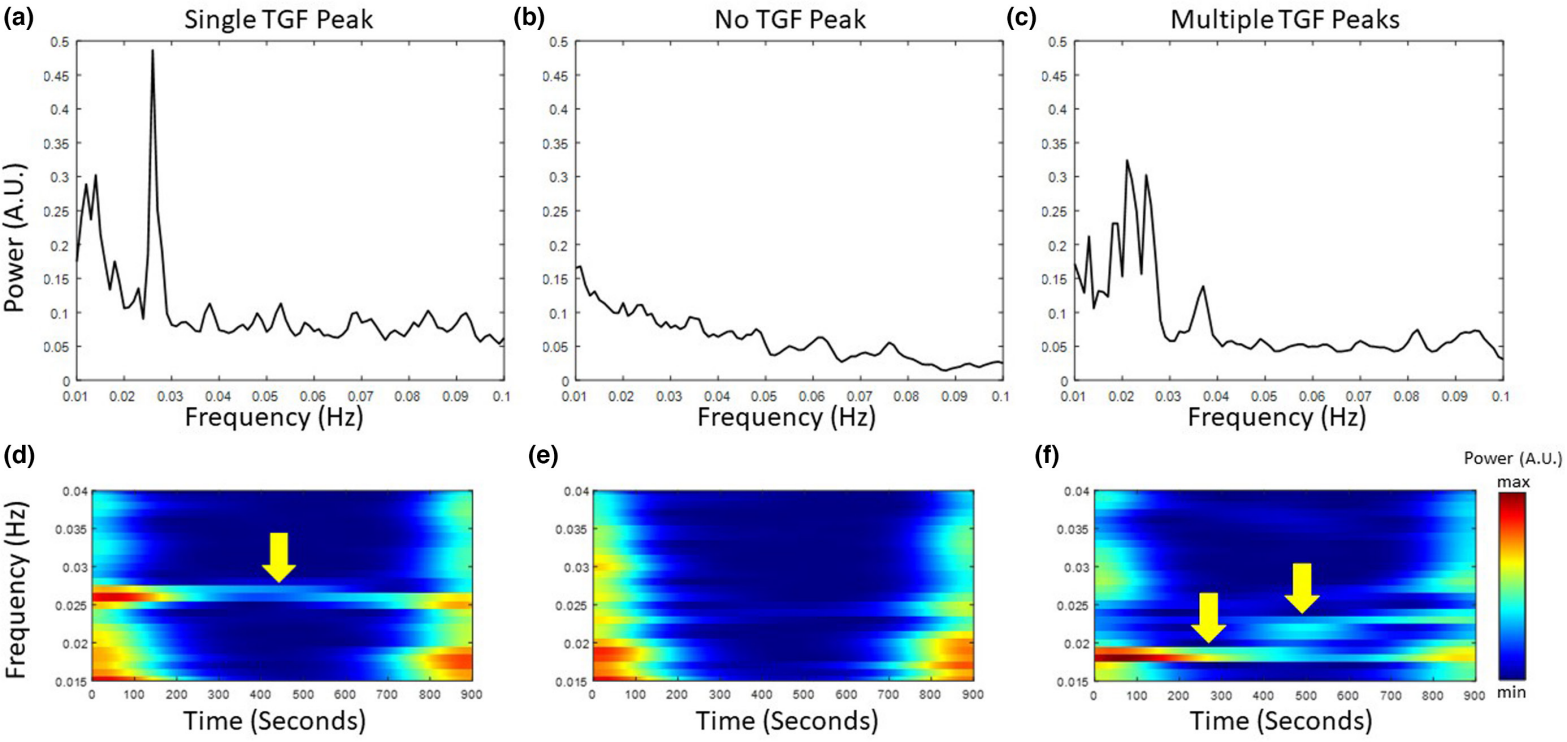
Three types of TGF‐induced responses in different vessels. The top panel represents time‐averaged power spectra: One peak around 0.03 Hz corresponds to a single frequency of TGF oscillation (a); a TGF peak cannot be detected (b); Multiple peaks within the narrow frequency band correspond to TGF oscillations with different frequencies (c). The bottom panel represents the time‐frequency spectrogram matching to power spectra on (a–c): A stable TGF frequency over the observation time (d), No observable TGF frequency (e); Multiple TGF frequencies within the observation time (f).

With the implementation of the superlet analysis, we explored how nephron hemodynamics can vary from vessel to vessel. Although TGF is known to be a persistent mechanism in the nephrons, we found three distinct types of hemodynamic behaviors in the power spectrum, shown in Figure 3. In some nephrons, TGF operates at a single frequency stable over the observation period (Figure 3a). In other nephrons, we did not see any TGF peaks in the power spectrum (Figure 3b). Although this does not mean the TGF mechanism does not exist, it may be indistinguishable from other dynamics and noise. In many cases, we found that nephrons can have dynamic TGF frequencies over time, exhibiting multiple peaks between 0.015 and 0.04 Hz (Figure 3c). For example, the time‐frequency spectrogram in Figure 3f shows that the TGF frequency slowly migrates from 0.02 to 0.025 Hz over the observation period of 900 s.

### 3.2 Acute effect of Na‐K‐2Cl co‐transporter inhibition on TGF dynamics

Furosemide, a loop diuretic that blocks Na‐K‐2Cl‐ co‐transporters, is known to cause diuresis and increase the excretion of sodium, chloride, and other ions. In animals receiving the systemic infusion of furosemide, urine flow increased from 12.6 ± 2.95 to 63.0 ± 3.63 μL/min (*p* < 0.01). The urinary sodium excretion increased from 0.38 ± 0.29 to 5.98 ± 0.57 μEq/min (*p* < 0.01). The blood pressure remained unchanged from 112.16 ± 3.21 to 108.49 ± 2.21 mm Hg.

**FIGURE 4:**
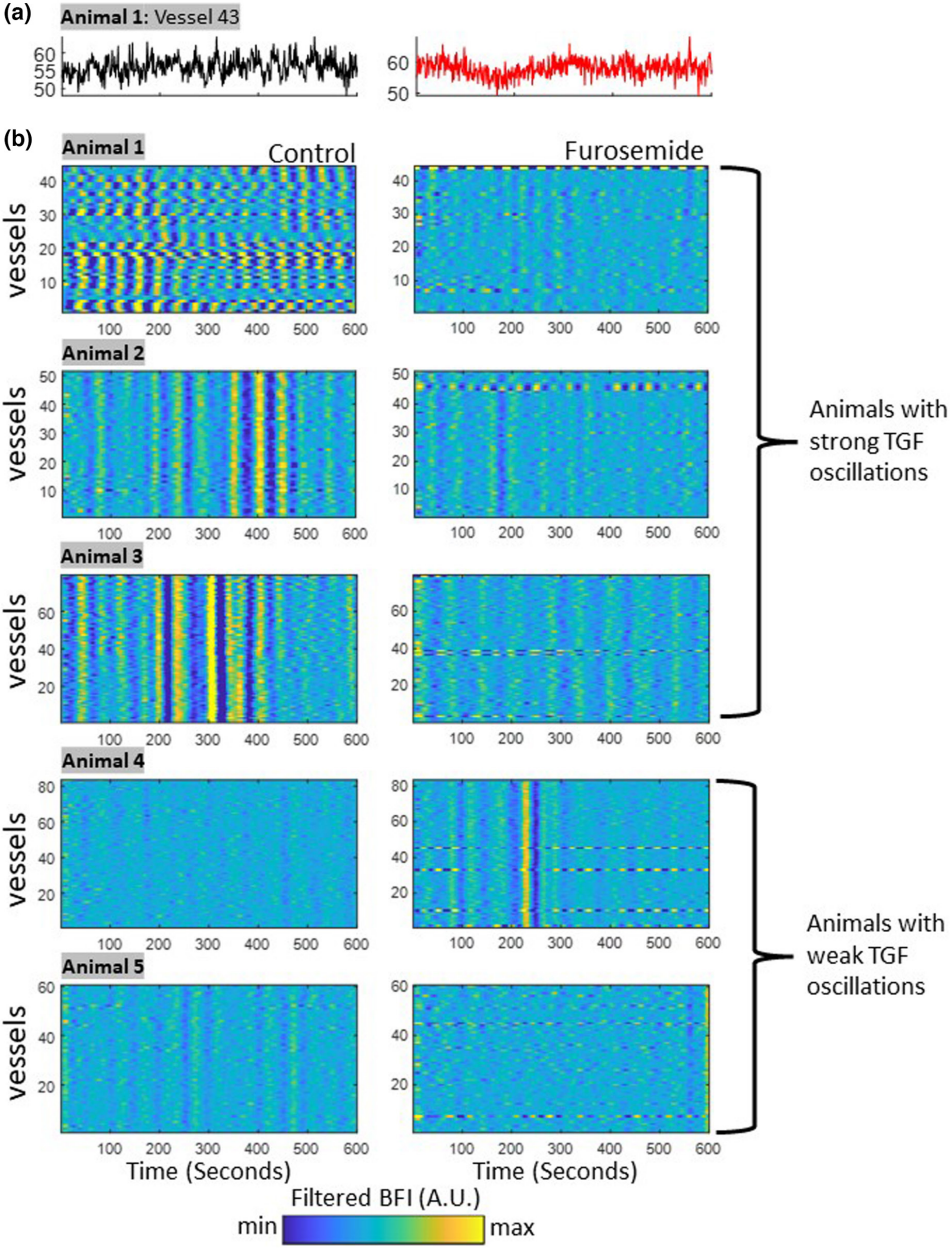
Eliminating TGF with furosemide administration in normotensive Sprague–Dawley rats. (a) An example time series taken from one vessel shows that the TGF oscillations present during the control period (black) disappear after furosemide infusion (red). (b) Carpet plots show TGF‐mediated blood flow oscillations across many vessels, with the color range representing filtered blood flow index. Oscillations are strong in animals 1–3 and weak in animals 4 and 5 in control (first column). Regardless, the TGF oscillations are eliminated in all animals, as demonstrated by the lack of oscillatory patterns (second column). The plots only show 600 s of 30‐min recordings to visualize the oscillatory patterns better.

With the systemic infusion of furosemide, we abolished the TGF mechanism and analyzed the hemodynamic changes in 317 microvessels associated with individual nephrons across five Sprague–Dawley rats. Figure 4 shows that animals 1, 2, and 3 exhibited strong TGF‐mediated oscillations during the control period. Interestingly, vessels in animal 1 showed heterogeneous phases across the observed vessels, while animals 2 and 3 showed synchronized oscillations. The synchronizations are especially evident between 300 and 500 s in animal 2 and between 300 and 400 s in animal 3. After the furosemide infusion, the oscillations disappear, evidenced by the lack of oscillatory patterns in the carpet plot. The oscillations do not reach the minimum and maximum color values as they do in the carpet plots for control. Animals 4 and 5 showed weak TGF oscillations during the control period (compared to animals 1–3), and the lack of TGF‐mediated hemodynamics continued during the furosemide infusion period. Nevertheless, all animals show that the TGF oscillations have been eliminated after Na‐K‐2Cl inhibition despite having various amplitudes of TGF oscillations during the control period.

**FIGURE 5:**
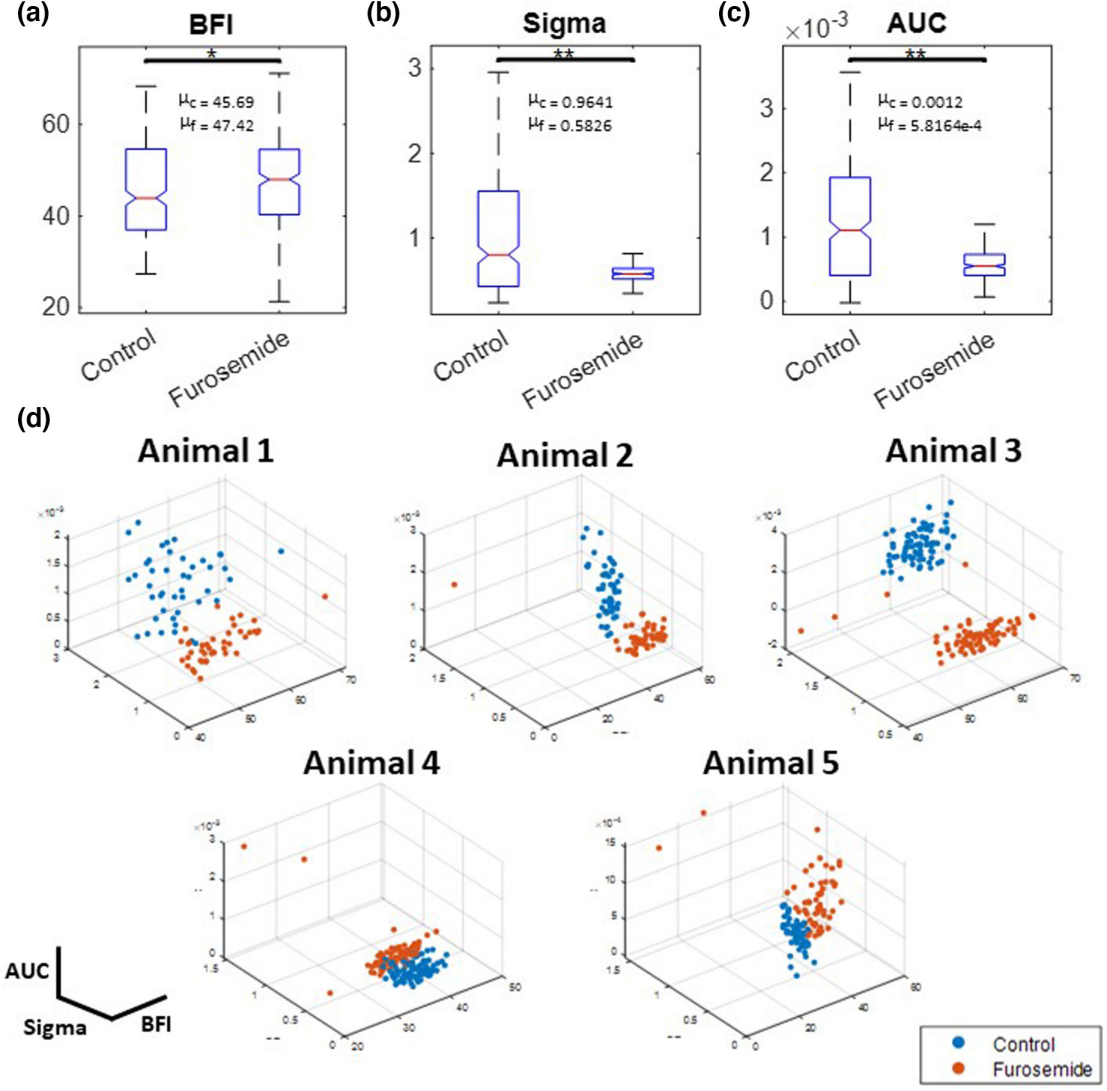
Increased local blood flow and the TGF dynamic changes induced by furosemide administration. Top panel: Box plots represent the median (central red line), the 25th (bottom edge), and 75th (top edge) percentiles of the analyzed TGF metrics: (a) mean blood flow index (BFI), (b) standard deviation of filtered TGF time series (Sigma), and (c) area under the curve (AUC). Reduced Sigma and AUC metrics represent the elimination of TGF oscillations. (d) Three metrics are plotted for every observed microvessel. Top row: 3 animals show a good separation in metrics between the control and the furosemide condition. Bottom row: Metrics of control and furosemide are clustered together. *p < 0.001; **p < 0.005. The units for BFI, Sigma, and AUC are arbitrary. ^μ^Mean values for control and furosemide data points.

To quantify the visual changes shown in Figure 4, we selected three metrics to compare between control and furosemide as described in the methods: mean blood flow index (BFI), the standard deviation of the TGF bandpass‐filtered signal (Sigma), and the area under the curve of the power spectrum (AUC) (Figure 5a–c). We found that furosemide had a significant effect on the metrics associated with the TGF: the BFI increased (*F* = 12.13, *p* < 0.001), while the Sigma and the AUC decreased significantly (*F* = 86.6, *p* < 0.0005; *F* = 226.2, *p* < 0.0005). A decrease in Sigma indicates the lack of TGF‐induced oscillations in the filtered signal. The reduced AUC shows that the power of the TGF frequency band in the power spectrum is weak. In Figure 5d, we used a 3‐dimensional scatter plot to show the spatial separation of the three metrics between control and furosemide for every segmented microvessels (*n* = 318). Animals 1, 2, and 3 show a similar pattern of separation in the metrics with minimal overlap in data points between the control and after the furosemide infusion. The spatial separation of the data points is less visible in animals 4 and 5 because they had weak TGF oscillations in control (Figure 5). Lastly, it should be noted that animal 5 seems to have a slightly higher AUC after furosemide relative to the control period, but this is a negligible difference. Details of the statistics are provided in Table S1.

### 3.3 Effect of acute sodium‐glucose co‐transporter 2 inhibition on TGF dynamics

We infused phlorizin, a sodium‐glucose co‐transporter inhibitor known to increase the urine flow and the excretion of sodium and glucose in the urine. We observed a significant increase in urine flow rate from 11.11 ± 2.83 to 24.17 ± 4.83 μL/min (*p* < 0.05). The glucose excretion increased from 0.01 to 4.77 ± 1.14 μEq/min (*p* < 0.05) along with increased sodium excretion from 0.56 ± 0.27 to 3.50 ± 0.35 μEq/min (*p* < 0.01). The blood pressure remained unchanged from 108.29 ± 5.58 to 100.73 ± 4.93 mm Hg.

**FIGURE 6:**
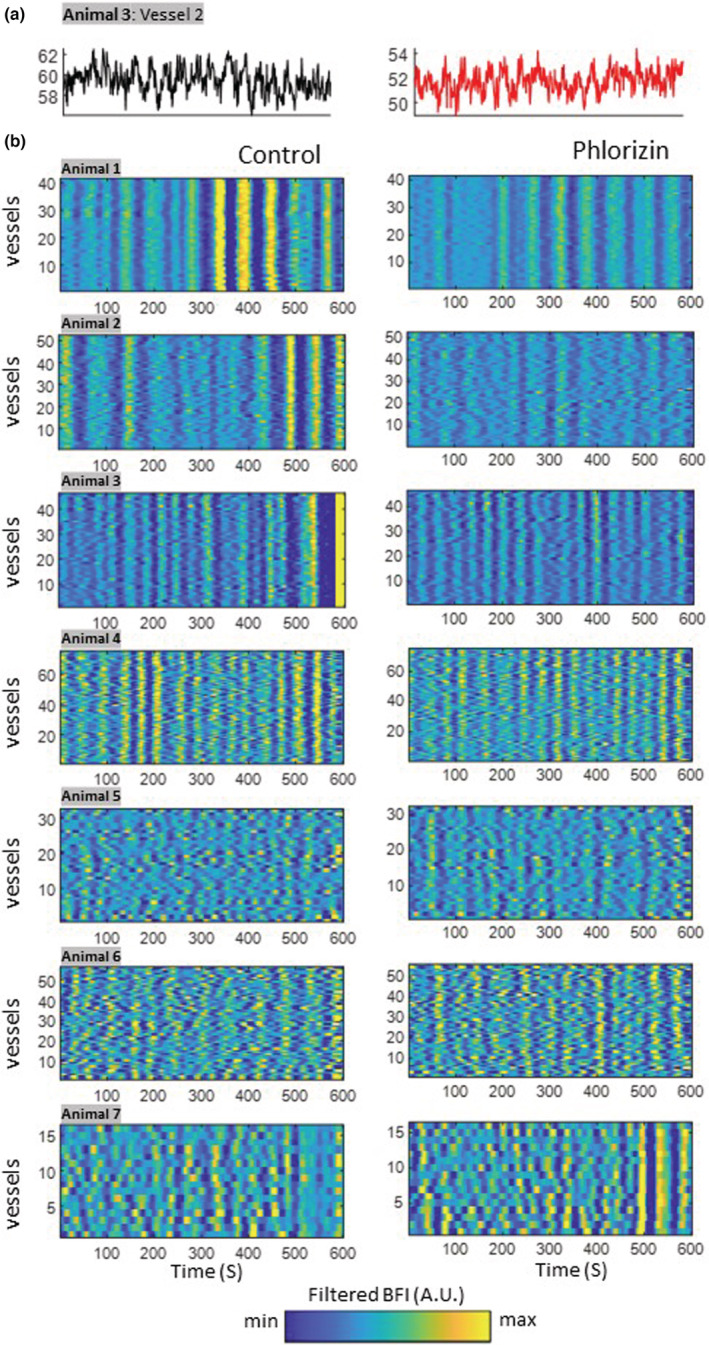
TGF oscillations in segmented vessels under phlorizin administration in normotensive Sprague–Dawley rats. (a) Exemplary blood flow time series demonstrating sustained TGF oscillations for 600 s in both control and phlorizin. Note the different *y*‐axis in the blood flow index (a.u.). (b) First column (control): TGF band‐filtered signals show consistent TGF oscillations over the observation time. In the second column (phlorizin): TGF oscillations are still well pronounced. The plots only show 600 s of 30‐min recordings to visualize the oscillatory patterns better. ^μ^Mean values for control and furosemide data points.

To explore the changes in TGF hemodynamics induced by inhibiting sodium‐glucose co‐transporter 2, we systemically administered phlorizin in rats and observed the renal hemodynamics. Hemodynamics in 318 microvessels associated with individual nephrons were analyzed across seven Sprague–Dawley rats. As expected, Figure 6a shows that TGF oscillations remain intact after the infusion but operate at a reduced blood flow. An exemplary blood flow time series shows TGF‐mediated oscillations around BFI (a.u.) of 60 during the control period that reduces to 52 during the phlorizin infusion while maintaining the oscillation. During the control period, animals 1–4 show some degree of synchronization in TGF oscillations across vessels, which can be interpreted from the vertical blue‐yellow striations. Animals 5, 6, and 7 show weak synchronicity in TGF oscillations, evidenced by the lack of vertically aligned striations in their respective carpet plots: the phase and frequency of the TGF oscillations vary across the observed vessels. Overall, animals show different levels of oscillatory synchronicity during the control period. After the infusion of phlorizin, the TGF oscillations are maintained, regardless of the degree of synchronization. The phlorizin plots do not reach the maximal color intensity compared to the control plots (Figure 6b), indicating a reduction in the blood flow index.

**FIGURE 7:**
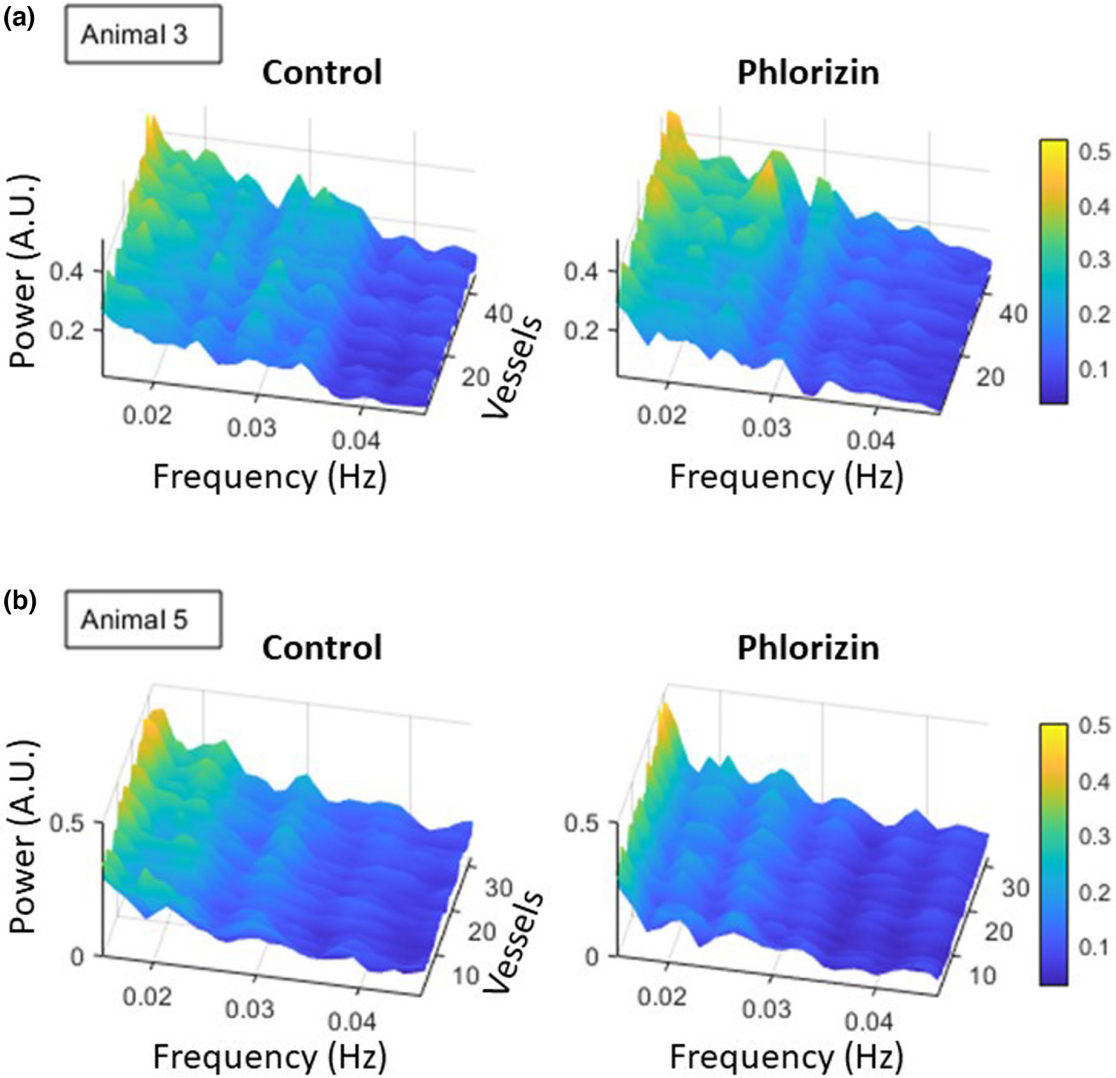
Different vessels can exhibit different amplitudes of the TGF frequency peak. (a) The time‐averaged power spectra of 40+ vessels show that vessels can have heterogeneous TGF peak amplitudes during the control period. After the phlorizin infusion, the dominant TGF frequency peak around 0.03 Hz remains well‐visible but exhibits variations in power across vessels. (b) Vessels show a dominant TGF frequency between 0.025 and 0.03 Hz in varying degrees of power in both conditions.

The diversity in TGF‐driven oscillatory patterns across vessels in an animal can be further understood by looking at Figure 7. The vessels show varying dominant TGF frequencies in the collective time‐averaged power spectra. More importantly, the power of the TGF frequencies varies across vessels. It was previously mentioned that animal 3 showed synchronized TGF oscillations (strong vertical striations) while animal 5 showed weak synchronization (no vertical striations) in Figure 6 during the control period. Regardless of the level of synchronicity, Figure 7 shows that the magnitude of TGF‐induced oscillatory hemodynamics can differ from vessel to vessel within an animal. After the phlorizin infusion, the diversity in the power of TGF frequency still exists, although the dominant TGF frequency may vary.

**FIGURE 8:**
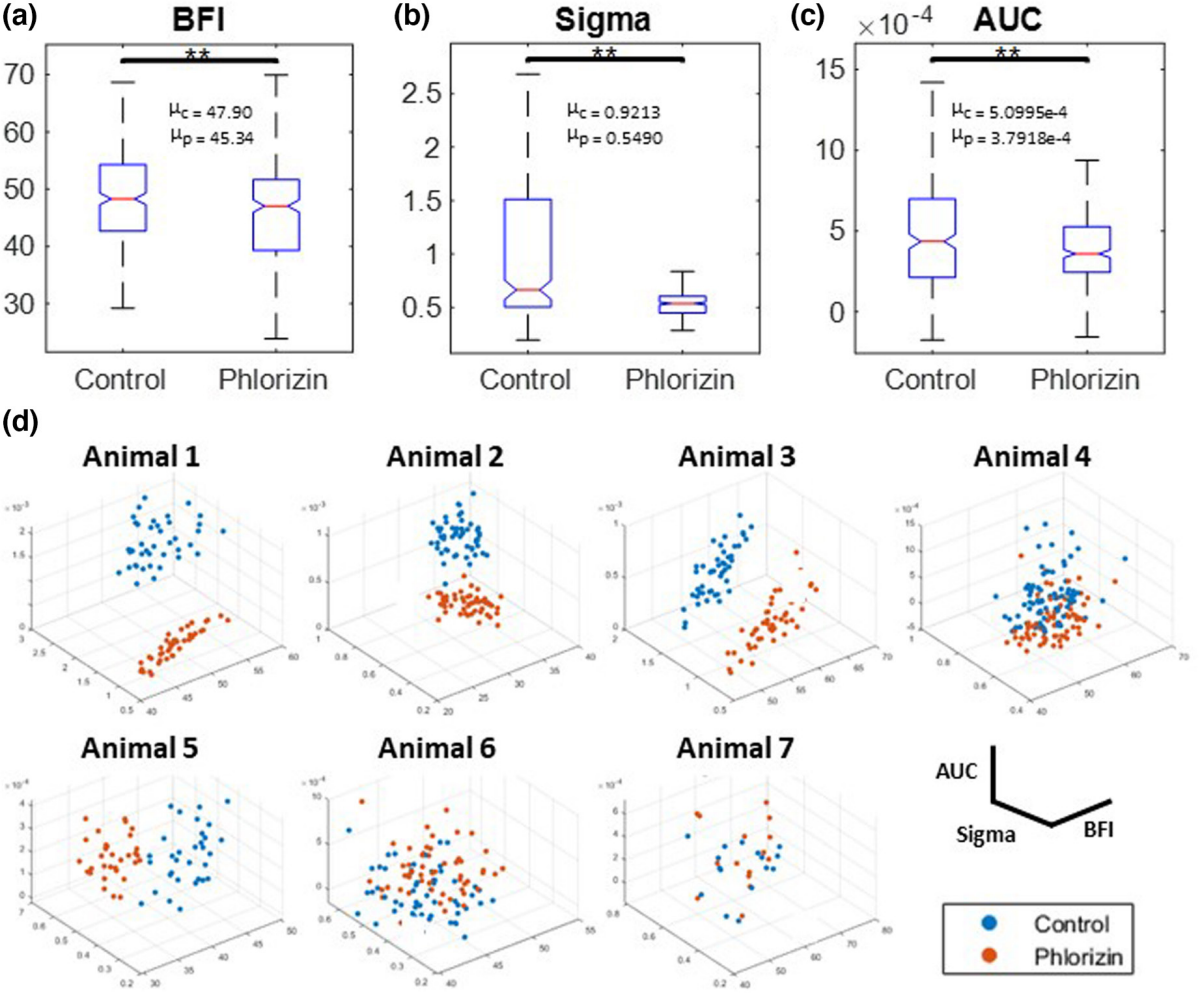
Decreased nephron blood flow and TGF dynamic changes induced with phlorizin administration. Top panel: Box plots represent the median (central red line), the 25th (bottom edge), and 75th (top edge) percentiles of the analyzed TGF metrics: (a) mean blood flow index (BFI), (b) standard deviation of filtered TGF time series (Sigma), and (c) area under the curve (AUC). (d) Three‐dimensional scatter plot of the three metrics for each microvessels. (d) Top row: 3 animals show a good spatial separation across all three dimensions. Bottom row: control and phlorizin clusters show similar metrics in 2 animals. **p < 0.0005. The units for BFI, Sigma, and AUC are arbitrary. ^μ^Mean values for control and phlorizin data points.

We quantified the same metrics for the phlorizin group as we did for the furosemide group (Figure 5) to measure the altered TGF‐mediated hemodynamics in individual vessels induced by phlorizin. We observed a significant decrease in the BFI (*F* = 48.4, *p* < 0.0005), presumably associated with increased afferent arteriolar resistance in observed nephrons (Figure 8a). A significant decrease in Sigma (*F* = 274.6, *p* < 0.0005) indicates a smaller amplitude for TGF oscillations. The AUC was also reduced (*F* = 132.3, *p* < 0.0005), indicating a weaker contribution of the TGF oscillation to the nephron hemodynamic partially caused by a smaller sigma. Figure 8d shows that the data points plotting the metrics per vessel separate the control from the intervention period well. Animals 1–4 show an apparent reduction in the AUC and the sigma across all imaged microvessels, indicating a change in the TGF‐mediated hemodynamics induced by phlorizin. Although animal 5 shows a minimal change to the signal and the AUC in vessels, there is a prevalent decrease in the BFI. Animals that originally had weak TGF oscillations (animals 6 and 7) show considerable overlap in data points. Details of the statistics are provided in Table S1.

## 4 DISCUSSION

This study reveals altered TGF‐driven hemodynamics induced by furosemide and phlorizin in a large population of nephrons for the first time. The current paradigm considers TGF as a single‐nephron event, and the micropuncture technique remains a gold standard for answering questions in renal pathophysiology. Micropuncture methods can access the TGF‐induced dynamics at a single‐nephron resolution, which is suitable for establishing the connection between tubular NaCl load and tubular pressure (Vallon, 2009). However, this study contends that single‐nephron measurements are insufficient in understanding nephron hemodynamics by demonstrating the inherent diversity in TGF‐driven hemodynamics across vessels, time, and animals, highlighting the need for real‐time access to hemodynamics in a population of nephrons.

Here, we propose to look at TGF‐induced cortical hemodynamic responses through high‐resolution LSCI. This technique allows us to simultaneously access the TGF‐mediated vascular responses in many vessels on the kidney cortex. We show that administering furosemide increases the local microcirculatory blood flow by 11.3%, and the TGF oscillations become eliminated. There is a strong link between the changes in the predistal tubular reabsorption of solutes like sodium and glucose and the sequential perturbation of the TGF mechanism, which may alter the glomerular filtration pressure. An expected result of this study is increased microvascular blood flow by abolishing the TGF mechanism with furosemide. This paper's results are consistent with single‐nephron studies, which found that an intra‐luminal microperfusion of furosemide can abolish TGF‐mediated oscillations in Sprague–Dawley rats (Leyssac, 1986; Leyssac & Holstein‐Rathlou, 1986). The effect of furosemide on the TGF‐induced hemodynamics found in this study is obvious. But novelty lies in demonstrating the hemodynamical response associated with a population of nephrons with the superlet analysis‐ with improved sensitivity to the transient signals and SNR compared to other time‐frequency analyses (Moca et al., 2021).

To discuss the results of the phlorizin experiment, we distinguish between the TGF mechanism and the TGF‐mediated oscillations. While we expect the TGF mechanism to remain operable at all times, the TGF‐induced oscillations are temporally dynamic. Contrary to the effects of furosemide, phlorizin administration induces a relative drop in local microcirculatory blood flow by 6.42% and weakens (but sustains) TGF‐mediated oscillations in microvessels associated with nephrons. Previously, it was shown that SGLT2 inhibitors could reduce the glomerular capillary pressure via the afferent arteriolar constriction by eliciting the TGF (Ehrenkranz et al., 2005; Ghezzi et al., 2018; Sen & Heerspink, 2021; White Jr, 2010). It was also shown that dapagliflozin saturates the TGF response in early diabetic rats (Thomson, 2012). Plus, inhibiting proximal HCO3 reabsorption‐ similar to SGLT2 inhibition can reduce overall proximal reabsorption. Sequentially, the TGF can reset to a lower operating point when the proximal reabsorption is reduced, demonstrating the adaptability of TGF in response to fluid‐content changes (Deng et al., 2002; Scott et al., 1997). Furthermore, Kidokoro et al. (2019) showed that administering empagliflozin for 30 min in mice reduced the afferent arteriolar diameter in vivo. These accumulating studies provide a plausible explanation for the smaller oscillatory amplitudes (sigma) and the AUC seen in our results. Interestingly, there is an increased expression and activity of SGLT2 in diabetic kidneys, leading to a higher potency of SGLT2 inhibitors (Vallon & Thomson, 2017). Expanding this study to diabetic rat kidneys may unveil novel TGF‐driven hemodynamical patterns in response to SGLT2 inhibitors: We could potentially understand its real‐time nephroprotective influence on a compromised kidney.

This study also presents the observed variability in the dominant TGF dynamic among animals. Five out of 12 animals showed weak TGF oscillations in control (two from the furosemide group and three from the phlorizin group). These animals underwent the same surgical and experimental protocol, yet the TGF‐mediated hemodynamic diversity persisted. Similar observations were made in a study by Scully et al. (2014) in which a strong MR signal was detected in three out of six long Evans rats. The size of the imaged region is approximately 1700 × 1700 μm of the renal surface, and the weak TGF signal of segmented microvessels does not represent the whole kidney hemodynamics. Figure S1 demonstrates that the TGF signal exists during control in all animals in varying dominant frequency and power when the wavelet transform is applied to the blood flow time series of the whole renal surface using the low zoom data.

Several factors can be addressed for future studies. First, the group size could be expanded to make more robust statistical conclusions about hemodynamical changes induced by various tubular transporter inhibitors. Plus, including female rats in subsequent studies would mitigate the current limitation of this study. Second, a chronic study of an analog of phlorizin with fewer side effects would be more clinically relevant. Third, while this study does not quantify the network behavior of the nephrons, a phase‐frequency cluster analysis could be implemented to quantify the synchronicity level among nephron‐associated microvessels. This could reveal the collective behavior of nephron ensembles adapting to altered TGF‐induced hemodynamics. Finally, the pressure‐induced (myogenic) vasomotion and the TGF contribute to efficient autoregulation by modulating the afferent arteriolar tone (Carlström et al., 2015; Chon, 2005; Just, 2007). In spontaneously fluctuating single‐nephron blood flow obtained from Sprague–Dawley rats, there exists a slow oscillation (20–30 mHz) mediated by the TGF and a fast oscillation (100 mHz) connected to the myogenic activity (Chon, 2005). It was also shown that the TGF modulates the myogenic activity (Marsh, 2005), and myogenic oscillations are enhanced when TGF was inhibited with furosemide (Yip et al., 1993). Although this focuses on the TGF hemodynamics, it would be interesting to see if the myogenic mechanism can compensate for the reduced TGF oscillations observed after the inhibition of SGLT2. The ramifications of reduced TGF fluctuations are unclear: Is this a sign of renal decline or a mechanism the kidney uses to respond to a physiological imbalance?

Recent experiments showed that many nephrons coordinate their TGF‐induced hemodynamic responses, which can also be seen in the control data of our results (Figures 4 and 6). The presence of TGF oscillations links to the synchronization of vascular responses of neighboring nephrons (Brazhe et al., 2014; Holstein‐Rathlou et al., 2011; Mitrou et al., 2015). Recently, Postnov et al. revealed that renal microcirculatory blood flow tends to demonstrate clustered, frequency‐locked activity (Postnov et al., 2022). One could speculate that renal autoregulation provides better protection when nephrons act together. Cooperating nephrons can increase the efficiency of renal autoregulation by collectively engaging in more preglomerular resistance. Yet, the efficacy of such cooperative behavior on overall renal autoregulation remains an open question, and this paper's approach opens doors to answering these questions.

## CONFLICT OF INTEREST STATEMENT

The authors declare no competing interest.

## ETHICAL STATEMENT

The study was conducted in accordance with the Danish National Animal Experiments Inspectorate (protocol #: 2020‐15‐0201‐00547).

## Supporting information


Figure S1.
Table S1.
