# Supplementary material for: The assessment of cortical hemodynamic responses induced by tubuloglomerular feedback using in vivo imaging
Source: Physiol Rep. 2023 Mar 22;11(6):e15648. doi: 10.14814/phy2.15648 (PMC10034006; doi:10.14814/phy2.15648)
Supplement: Supplementary file 1 — Figure S1. Table S1. [file PHY2-11-e15648-s001.docx]

| Furosemide group LMM results | | | | | | | | |
| --- | --- | --- | --- | --- | --- | --- | --- | --- |
|  | Estimate | SE | tstat | DF | pValue | Lower | Upper | LogLik |
| BFI | 1.4813 | 0.4252 | 3.4831 | 632 | 0.0005 | 1.6462 | 2.3164 | -1977.1 |
| SD | -0.3079 | 0.0331 | -9.3043 | 632 | 2.1802e-19 | -0.3729 | -0.2529 | -354.36 |
| AUC | -0.0006 | 4.228e-5 | -15.04 | 632 | 7.6858e-44 | -0.0007 | -0.0006 | 3818.7 |
| Phlorizin group LMM results | | | | | | | | |
|  | Estimate | SE | tstat | DF | pValue | Lower | Upper | LogLik |
| BFI | -2.3819 | 0.3424 | -6.9563 | 634 | 8.7233e-12 | -3.0542 | -1.7095 | -1853.3 |
| SD | -0.3368 | 0.0203 | -16.572 | 634 | 1.6249e-51 | -0.3767 | -0.2969 | -54.655 |
| AUC | -0.0002 | 1.9966e-5 | -11.501 | 634 | 6.1675e-28 | -0.0003 | -0.0002 | 4351.4 |

Table S1: Linear mixed model results for TGF hemodynamic metrics


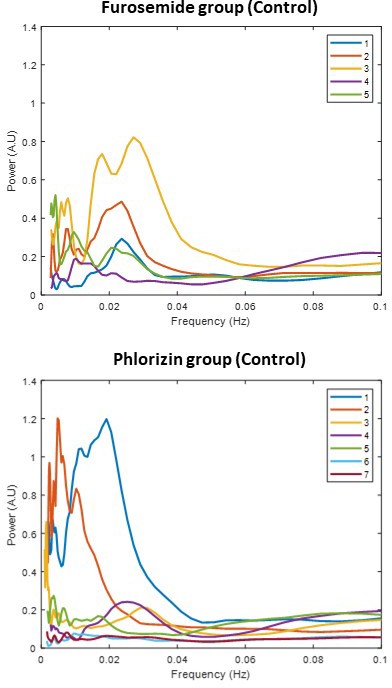


Figure S1: Power spectra of low zoom renal cortex blood flow time-series during the control period for (top) the furosemide group and (bottom) the phlorizin group. Both groups exhibit varying degrees of TGF frequency.
